# Aflatoxin exposure *in utero* and birth and growth outcomes in Tanzania

**DOI:** 10.1111/mcn.12917

**Published:** 2019-12-11

**Authors:** Simone Passarelli, Sabri Bromage, Anne Marie Darling, Jia‐Sheng Wang, Said Aboud, Ferdinand Mugusi, Jeffrey K. Griffiths, Wafaie Fawzi

**Affiliations:** ^1^ Department of Nutrition Harvard T.H. Chan School of Public Health Boston Massachusetts; ^2^ Department of Global Health and Population Harvard T.H. Chan School of Public Health Boston Massachusetts; ^3^ Department of Environmental Health Science University of Georgia Athens Georgia; ^4^ Department of Microbiology and Immunology Muhimbili University of Health and Allied Sciences Salaam Tanzania; ^5^ Department of Internal Medicine Muhimbili University of Health and Allied Sciences Salaam Tanzania; ^6^ Department of Public Health and Community Medicine Tufts University School of Medicine Boston Massachusetts; ^7^ Department of Epidemiology Harvard T.H. Chan School of Public Health Boston Massachusetts

## Abstract

Some evidence suggests that aflatoxin may contribute to the high prevalence of stunting observed in low‐income countries. Whereas several studies have been conducted in West Africa, fewer exist in East Africa and even fewer in nonagricultural contexts. We analyzed serum samples from 400 iron‐replete, nonanemic pregnant women from a cohort in Dar es Salaam, Tanzania to determine the extent and magnitude of exposure to aflatoxin and to study the relationship between levels of aflatoxin exposure in utero and infant birth and growth outcomes. Ninety‐nine percent of women had detectable concentrations of aflatoxin B1‐lysine (AFB1‐lysine), with a median level of 1.4‐pg/mg albumin, indicating a much lower level compared to studies of rural populations in sub‐Saharan Africa. Our results do not show a statistically significant relationship between AFB1‐lysine levels and birth weight, small for gestational age, or prematurity. We observe a small statistically significant reduction in gestational age at delivery (0.47 weeks; 95% CI: −0.86, −0.07) as the natural log of AFB1‐lysine levels increases by 1 unit of pg/mg of albumin, after controlling for potential confounders. Among a nonrandom set of infants who had measurements for placental weight, haemoglobin at delivery, and follow‐up z‐score measurements, we find no association between aflatoxin plasma concentrations and these variables. These findings suggest a high prevalence of chronic low‐level exposure to aflatoxin, though its effect on birth outcomes in this population remains unclear. Our research adds to a growing body of literature finding mixed associations between aflatoxins on pregnancy outcomes and child growth.

**Key messages**

- Previous research suggests that aflatoxin exposure may contribute to growth faltering among children in low‐income settings.
- We analyzed serum samples from 400 pregnant women from Dar es Salaam, Tanzania to observe the relationship between levels of aflatoxin exposure in utero and subsequent maternal and child growth outcomes.
- Aflatoxin B1‐lysine was detected in 99% of pregnant women, with a median of 1.4 pg/mg albumin—a relatively low concentration compared with other sub‐Saharan African studies.
- We did not observe a relationship between aflatoxin levels and growth. Aflatoxin exposure had a small, statistically significant negative association with gestational age at birth.

## 1 INTRODUCTION

Aflatoxins are a family of potent fungal toxins produced by the fungi *Aspergillus flavus* and *Aspergillus parasiticus* that contaminate soil, vegetation, and stored crops (Kitya, Bbosa, & Mulogo, 2010). They are found on a variety of staple crops including maize and groundnut. Aflatoxins thrive in the absence of adequate food safety measures and in the presence of crop damage and climatic stress. Consequently, aflatoxin contamination is highly prevalent in tropical and subtropical low‐income countries, particularly in sub‐Saharan Africa and southeast Asia (Gnonlonfin et al., 2013; Williams et al., 2004). Humans are exposed to aflatoxins through the ingestion of contaminated crops, through the consumption of products from exposed animals, or through vertical transmission in utero or in breast milk (Denning, Allen, Wilkinson, & Morgan, 1990). In addition to their acute hepatotoxic and immunosuppressive effects, cumulative long‐term exposure to aflatoxins has been associated with liver cancers, disturbances in the absorption and metabolism of several vitamins and minerals, adverse pregnancy outcomes, and growth faltering (Paul Craig Turner, 2013). The health implications of aflatoxin contamination are expected to become increasingly significant as climate change allows for more favourable conditions for aflatoxin biosynthesis in certain settings, and population growth continues to increase the demand for food production (Baranyi, Kocsubé, & Varga, 2015; Medina, Rodriguez, & Magan, 2014).

Among the most concerning aspects of widespread aflatoxin contamination in developing countries is its potential effect on the impairment of child growth, which is associated with lifelong cognitive and physical deficits (Dewey & Begum, 2011; Hack, Klein, & Taylor, 1995). Aflatoxin has been implicated as a contributing factor to stunting due to the overlap of aflatoxin exposure with stunting in Africa and Asia (Kaaya & Warren, 2005). The relationship between growth failure and aflatoxin exposure has long been seen in animals and has been increasingly observed in humans. Several reviews have been conducted on the relationship between aflatoxin exposure and growth faltering, synthesizing evidence of aflatoxin exposure associated with growth impairment in pigs, turkeys, quail, ducks, tilapia, and cows, in addition to a growing body of evidence for humans (Gnonlonfin et al., 2013; Khlangwiset, Shephard, & Wu, 2011; Shuaib, Ehiri, Abdullahi, Williams, & Jolly, 2010; Smith, Prendergast, Turner, Humphrey, & Stoltzfus, 2017; Paul Craig Turner, 2013; Williams et al., 2004). Children are more susceptible to aflatoxin exposure and toxicity, and aflatoxin may affect child growth in and ex utero through a range of mechanisms including hepatotoxic pathways, immunosuppression leading to acute and chronic infections like malaria and HIV, poor pregnancy outcomes including placental abnormalities and preterm birth, and through multifarious effects on maternal and child nutrition including disruption of iron status and anaemia (Khlangwiset et al., 2011; Paul C. Turner, Moore, Hall, Prentice, & Wild, 2003; Paul Craig Turner, 2013). In Gambian infants of aflatoxin‐exposed women, genome‐wide deoxyribonucleic acid methylation studies revealed significant aflatoxin‐associated differential methylation in 71 CpG sites. These included growth factor genes (FGF12, IGF1) and immunity‐related genes such as CCL28, TLR2, and TFGB1 (Hernandez‐Vargas et al., 2015). Thus on a molecular level, aflatoxin toxicity in infants may be mediated in part through early epigenetic programming.

Few studies have been conducted in urban settings in sub‐Saharan Africa, where exposure to contaminated crops may remain high despite their separation from agricultural contexts. Sub‐Saharan Africa is affected by the second highest prevalence of low birth weight (<2,500 g) globally (13%; World Health Organization, 2014) and the second highest regional prevalence of child stunting (34%; World Health Organization, 2017). Moreover, the majority of research investigating the effects of exposure in utero on birth and child growth outcomes has been conducted in West rather than East African settings. In this analysis, we examined the relationship between maternal levels of aflatoxin exposure in utero and growth and birth outcomes in infants in Dar es Salaam, Tanzania. To our knowledge, ours is the first study assessing these relationships in an urban East African population using a longitudinal approach.

## 2 METHODS

### 2.1 Study design

This study utilized stored blood samples collected as part of a randomized clinical trial of iron supplementation in Dar es Salaam, Tanzania. Participants in the previous study included primigravidae or secundigravidae pregnant women presenting at three large antenatal clinics from September 28, 2010, through October 4, 2012, uninfected with HIV at or before 27 weeks of gestation, not severely anaemic or iron deficient, and who intended to stay in Dar es Salaam until 6 weeks post‐partum. Upon enrollment, baseline blood samples were collected; 1,500 women were randomized to 60 mg per day of iron supplementation or placebo and were assessed at delivery for birth outcomes, hematologic parameters and iron status. Trained research nurses also assessed infant anthropometry at delivery. Of the 1,500 women, 96 gave birth to low birth weight infants (<2,500 g), and iron supplementation was not significantly associated with infant birth weight or incidence of low birth weight (Etheredge et al., 2015).

For the analysis presented here, 400 stored plasma samples were selected randomly from the 685 primigravid participants with singleton pregnancies who gave birth to live infants and had a stored baseline sample. This random sample was selected due to financial limitations of the laboratory analysis. Of the 685 samples, 57 could not be located or did not have sufficient volume for the analysis. Four hundred were randomly selected from these remaining 628 samples. One observation was excluded from the statistical analysis due to a missing birthweight, resulting in a final sample of 399 infants.

### 2.2 Analysis of plasma AFB1‐lysine

The samples used for this analysis were stored at −80°C for between 3 and 6 years in Dar es Salaam. An aliquot of each sample was removed from storage in Tanzania, packed in dry ice, and shipped in insulated containers to the laboratory in Athens, GA. All plasma samples were coded with an identification number and, upon receipt, were coded with a separate laboratory designated sample ID number. Plasma samples were analyzed with a high performance liquid chromatography (HPLC)‐fluorescence method, including measurement of albumin and total protein concentrations for each sample, digestion with protease to release amino acids, concentration and purification of the aflatoxin B1 (AFB1)‐lysine adduct, and finally separation and quantitation by HPLC (G. Qian, Tang, Liu, & Wang, 2010; Guoqing Qian, 2012; Guoqing Qian et al., 2013). Specifically, thawed plasma samples were inactivated for possible infectious agents via heating at 56°C for 30 min, followed by measurement of albumin and total protein concentrations using modified procedures as previously described. A portion of each sample (approximately 150 μL) was digested by pronase (pronase: total protein, 1:4, w:w) at 37°C for 3 hr to release AFB1‐lysine. AFB1‐lysine in digests was further extracted and purified by passing through a Waters MAX SPE cartridge, which was preprimed with methanol and equilibrated with water. The loaded cartridge was washed with water, 70% methanol, and 1% ammonium hydroxide in methanol at a flow rate of 1 ml/min. Purified AFB1‐lysine was eluted with 2% formic acid in methanol. The eluent was vacuum‐dried with a Labconco Centrivap concentrator (Kansas City, MO) and reconstituted for HPLC‐fluorescence detection.

The analysis of AFB1‐lysine adduct was conducted in an Agilent 1200 HPLC‐fluorescence system (Santa Clara, CA). The mobile phases consisted of buffer A (20‐mM NH_4_H_2_PO_4_, pH 7.2) and buffer B (100% methanol). The Zorbax Eclipse XDB‐C18 reverse phase column (5 micron, 4.6 × 250 mm) equipped with a guard column was used. Column temperature was maintained at 25°C during analysis, and a volume of 100 μL was injected at a flow rate of 1 ml/min. A gradient was generated to separate the AFB1‐lysine adduct within 25 min of injection. Adduct was detected by fluorescence at maximum excitation and emission wavelengths of 405 and 470 nm, respectively. Calibration curves of authentic standard were generated weekly, and the standard AFB1‐lysine was eluted at approximately 13.0 min. Quality assurance and quality control procedures were taken during analyses, which included simultaneous analysis of one authentic standard in every 10 samples and two quality control samples daily. The limit of detection was 0.1‐pg/mg albumin, and the limit of quantitation was 0.4‐pg/mg albumin, based on the use of spiked authentic standard to 150‐μL plasma sample. The average recovery rate was 90% calculated from spiked samples with a low (1 pg) and a high (50 pg) authentic AFB‐lysine adduct. The AFB1‐lysine concentration was adjusted by albumin concentration for the report.

This method has been used to measure blood aflatoxin concentrations for over 20 years and has been applied in several studies to investigate the link between AF exposure and human health effects (Sun et al., 2002; J S Wang et al., 1996; Jia Sheng Wang et al., 2001). It is highly correlated with both enzyme‐linked immunosorbent assay (ELISA) and mass spectrometry, is appropriate for describing long‐term aflatoxin status (McCoy et al., 2008), and provides a range that is appropriate for measuring blood aflatoxin concentrations lying in the range of prior studies in East African populations. The AFB1‐lysine adduct has been shown to be stable for decades in human serum stored at −80°C (Scholl & Groopman, 2008); the samples used in this study were collected between 2010 and 2012 and maintained at this temperature.

### 2.3 Statistical analyses

The primary exposure of interest was aflatoxin B1 levels in pregnant women, based on plasma samples collected at enrollment. Four observations with AFB1‐lysine concentrations below the limit of quantitation (0.4 pg/mg of albumin) were replaced with the value of 0.4. The natural log‐transformed AFB1‐lysine values were used as the exposure variable for all regression analyses to improve the normality of the residuals.

Several maternal and infant outcomes were analyzed based on hypothesized associations in the literature between these variables and aflatoxin concentrations in utero: the birth weight of the child in kilogrammes; a binary indicator for whether the child was small for gestational age (SGA) using INTERGROWTH standards, defined as a weight for gestational age below the 10th percentile (Villar et al., 2014); gestational age at delivery; haemoglobin levels of the mother at birth; low birth weight, defined as less than 2,500 g; placental weight; and a binary variable for whether the child was premature, defined as a delivery earlier than 37 weeks of gestation. Gestational age was defined based on the date of last menstrual period, as reported by participants during the enrollment visit. For a subsample of children who had follow‐up visits with anthropometric measures (mean follow‐up time for those with weight = 46 days; range = 17–507; IQR = 25), we also examined the outcomes of length‐for‐age (*n* = 185), weight‐for‐age (220), and weight‐for‐length (*n* = 183) z‐scores at follow‐up. Biologically implausible z‐scores with values greater than 6 or less than −6 standard deviations were excluded.

Logistic regression models were run for binary outcomes (SGA, low birth weight, and prematurity), whereas ordinary least squares regressions were run for continuous outcomes (birth weight; gestational age at birth; haemoglobin levels; length‐for‐age, weight‐for‐age, weight‐for‐length; and placental weight). Given considerable missingness in child anthropometry at follow‐up, and in placental weight and maternal haemoglobin concentrations at delivery, analyses of these outcomes are included in the supplemental tables to this paper, because these subsamples may not be representative of our study population.

Control variables were included that have established confounding relationships in the literature or that had possible bivariate associations (*p* < .25) with our primary outcome of interest. We adjusted for the treatment regimen (iron vs. placebo), though this is expected not to be a confounder because it was randomized and thus unassociated with aflatoxin levels. We adjusted for socioeconomic status of the mother using wealth quintiles, age, employment category (skilled, unskilled/informal, or unemployed) and education level (a binary indicator for greater than or less than or equal to the median 8 years of education, calculated as binary due to low variation in this variable). Wealth quintiles were created through a principal component analysis, considering the first principle component and dividing the principal component analysis scores into quintiles (Rutstein & Rojas, 2006). The variables for the principal component analysis were selected based on the highest proportion of variation explained and included: possession of electricity, a generator, running water, a fan, a refrigerator, a car, a television, a radio, a couch, and a bicycle. Gestational age at delivery, baseline body *m*ass *i*ndex *(*BMI) of the mother, sex of the child, and maternal height were included due to their established relationship with the size of the child at birth.

We conducted several subset and sensitivity analyses. Due to the spread of our data, we had little power to observe relationships between aflatoxin exposure levels and birth outcomes among those with high levels of aflatoxin exposure. Because we were also interested in the effects of chronic low‐level aflatoxin exposure on our outcomes, we examined the same relationships presented in Table 3 in a subset of our data, for individuals with exposure levels under 5.0 pg/mg of albumin, because 95% of observations lie in this range, and the outliers were highly influential. These parameters excluded 21 woman–infant pairs. We also explored effect modification between aflatoxin levels and wealth, age, education, height, and baseline BMI of the mother, sex of the child, and gestational age at enrollment. We tested for a potentially nonlinear association between serum AFB1‐lysine concentration and gestational age at birth within the low AFB1‐exposure subset. Gestational age at birth was modeled as a generalized additive function (Wood, 2004) of AFB1 levels and all control variables, using regression splines for AFB1‐lysine and continuous control variables.

For our sensitivity analyses, we tested whether the effect of aflatoxin exposure on birth weight was mediated by gestational age at delivery. We also restricted our analysis to individuals in the upper and lower quartiles of AFB1‐lysine levels to observe whether there were any relationships between aflatoxin exposure and our outcomes that were only evident at the extremes. All data were analyzed using Stata 15.0 (all regression analyses aside from splines and descriptive graphs), SAS 9.4 (selection, data cleaning, and variable construction), and R statistical software (spline analysis and graphs).

### 2.4 Ethics statement

The study was conducted in compliance with the ethical principles of the Helsinki Declaration. Ethical approval was obtained from Institutional Review Board at the Harvard T.H. Chan School of Public Health and the Muhimbili University of Health and Allied Sciences Senate Research and Publications Committee.

## 3 RESULTS

### 3.1 Descriptive analyses

Table 1 displays general demographic and health characteristics of our sample, disaggregated by the median level of AFB1‐lysine and overall. A Student's *t*‐test for continuous variables and a proportion test for binary variables comparing individuals above and below the median level of exposure show no statistically significant differences in characteristics between these two groups (based on a *p* < .05). The two variables with *p*‐values below 0.2 show that women with low exposure are about 8 months younger compared with women with high exposure, and haemoglobin levels at baseline were approximately 0.2 g/dl lower among women in the high exposure group. Overall, we do not observe large differences in selected characteristics between those with of high versus low aflatoxin plasma concentrations in this bivariate test.

**Table 1:** Descriptive statistics of sample overall and by low versus high AF‐alb level

	Below median AF‐alb (1.39 pg/mg albumin)			Above median AF‐alb (1.39 pg/mg albumin)			Overall		
Variables	*N*	Mean	95% CI	*N*	Mean	95% CI	*p‐*value	Mean	95% CI
BMI at baseline, kg/m^2^	199	23.78	(23.21, 24.35)	200	24.06	(23.49, 24.63)	.49	23.92	(23.52, 24.32)
Maternal age, year	199	22.80	(22.27, 23.33)	200	22.17	(21.71, 22.62)	.06	22.48	(22.14, 22.83)
Birth weight, g	199	3.13	(3.06, 3.21)	200	3.13	(3.05, 3.21)	.84	3.13	(3.08, 3.18)
Gestational age at delivery, week	199	39.25	(38.87, 39.64)	200	39.09	(38.68, 39.51)	50	39.17	(38.89, 39.45)
Haemoglobin at baseline, g/dL	199	11.62	(11.44, 11.79)	200	11.78	(11.59, 11.96)	0.18	11.70	(11.57, 11.82)
Height of mother, cm	199	155.74	(154.95, 156.54)	200	156.48	(155.65, 157.30)	.22	156.11	(155.54, 156.68)
Birth weight <2,500 g	199	0.07	(0.04, 0.11)	200	0.06	(0.03, 0.10)	.83	0.06	(0.04, 0.09)
Premature (<37 weeks)	199	0.17	(0.12, 0.23)	200	0.16	(0.11, 0.21)	.72	0.16	(0.13, 0.20)
SGA, <3rd percentile	199	0.09	(0.05, 0.14)	200	0.07	(0.04, 0.11)	.45	0.08	(0.56, 0.11)
SGA, <10th percentile	199	0.20	(0.15, 0.26)	200	0.20	(0.14, 0.26)	.88	0.20	(0.16, 0.24)
Has electricity	199	0.25	(0.19, 0.31)	200	0.21	(0.15, 0.27)	.32	0.23	(0.19, 0.27)
Has running water	199	0.67	(0.60, 0.73)	200	0.62	(0.54, 0.68)	.27	0.64	(0.59, 0.69)
Woman is married	199	0.74	(0.68, 0.80)	200	0.77	(0.71, 0..83)	.54	0.76	(0.71, 0.80)
Years of education									
<4 or missing	199	0.07	(0.04, 0.11)	200	0.06	(0.03, 0.10)	.83	0.06	(0.04, 0.09)
5–7	199	0.48	(0.41, 0.55)	200	0.49	(0.41, 0.56)	.96	0.48	(0.43, 0.53)
8–11	199	0.27	(0.21, 0.34)	200	0.32	(0.25, 0.38)	.34	0.29	(0.25, 0.34)
12+	199	0.18	(0.13, 0.24)	200	0.14	(0.10, 0.20)	.27	0.16	(0.12, 0.20)

*Note*. The *p*‐value refers to the statistical significance of the *t*‐statistic is for the comparison of high and low AFB1‐lysine, calculated as a test of low–high, using a Student's *t*‐test assuming equal variances for continuous measures; binary measures (prematurity, SGA, electricity, married, water, and education categories) used a two‐sample proportion test with equal variances, and the associated *p*‐values refer to the statistical significance of the z‐statistic. Binary variables are reported as proportions and their confidence intervals are based on a binomial exact distribution; confidence intervals are based on the normal distribution for continuous variables.

Abbreviations: AFB1, aflatoxin B1; BMI, body mass index; SGA, small for gestational age.

**Figure 1:**
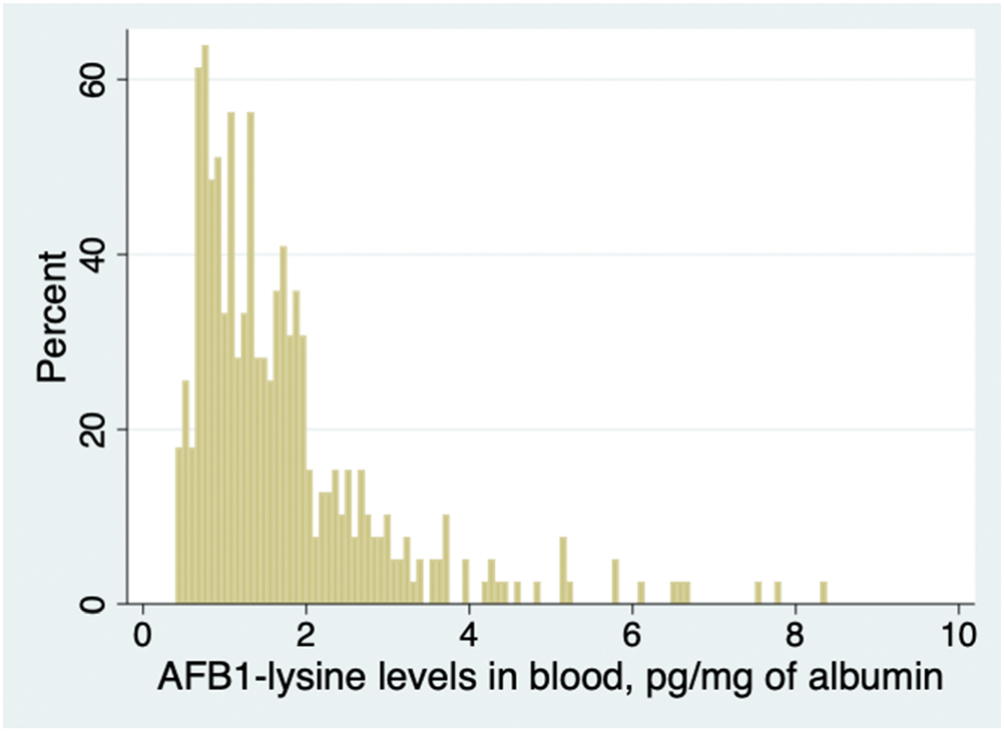
This histogram shows the distribution of the vast majority of observations, with aflatoxin B1‐lysine levels <10 pg/mg

Analysis of the 399 serum samples shows a 98.99% prevalence of detectable aflatoxin metabolites (Table 2, Figure 1). Only four observations had a result below the minimum detectable level of 0.4 pg/mg of albumin. The geometric mean in the population was 1.52‐pg/mg albumin (95% CI: 1.42, 1.64; range for detectable levels: 0.42–84.78). Figure 1 illustrates the spread of the data, zooming in on values of 10 pg/mg or below (seven observations were above this level and were omitted from the graph for readability purposes). The distribution has a pronounced right skew—approximately 95% of all observations have a value of 5 pg/mg or less.

**Table 2:** Sample AFB1‐lysine adduct levels

AFB1‐lysine (pg/mg albumin)	
Mean	2.41
Standard deviation	5.89
Median	1.40
Q1	0.92
Q3	2.10
Geometric mean	1.52
95% CI	(1.42, 1.64)
Minimum	0.42
Maximum	84.78

Abbreviations: AFB1, aflatoxin B1; Q1, first quartile; Q3, third quartile.

### 3.2 Regression analyses

Table 3 displays results from the linear and logistic regression analyses. We did not find any statistically significant relationships between aflatoxin plasma concentration and birth weight, size for gestational age, low birth weight, or SGA (<10th percentile). We also did not observe statistically significant associations between the exposure and more extreme indicators of these outcomes, including very low birth weight (<2 kg), SGA <3rd percentile, or prematurity <34 weeks (data not shown). Lastly, we find a small statistically significant reduction in gestational age at delivery (0.47 weeks; 95% CI: −0.86, −0.07) as the natural log of AFB1‐lysine levels increased by 1 unit of pg/mg of albumin. This equates to a 10% increase in AFB1‐lysine being associated with a reduction in gestation of 0.04 weeks—a very small effect. Supplemental analyses for which we had an incomplete sample investigated the outcomes of placental weight, haemoglobin concentration of mother at delivery, length‐for‐age, weight‐for‐age, and weight‐for‐length. Our data did not show statistically significant associations between these outcomes and the level of AFB1‐lysine at baseline (see 1).

**Table 3:** Multivariate regression analysis of the natural log of AFB1‐lysine levels on various birth outcomes

	Linear regression coefficients		Risk ratios from log‐binomial regression		
Variables	Birth weight, g	GA at delivery, weeks	SGA, <10th percentile	Low birthweight, <2,500 g	Premature, <37 weeks
ln of AFB1‐lysine, pg/mg albumin	−.03	−.47**	0.94	0.98	0.95
95% confidence interval	(−.10, .05)	(−.86, −.07)	(.73, 1.23)	(.60, 1.58)	(1.70, 1.30)
*p*‐value of coefficient	.48	.02	.66	.92	.74
Constant	1.02	35.44***	1.15	−18.94	0.50
Observations	399	399	399	399	399
*R* ^2^	.075	.066			

*Note*. Multivariate regressions control for asset quintile, employment category, age of mother, sex of child, education category, treatment regimen, baseline body mass index of mother, average height of mother across all prenatal appointments, and gestational age at enrollment. Coefficients and 95% confidence intervals displayed are risk ratios for SGA, low birthweight, and prematurity.

Abbreviations: AFB1, aflatoxin B1;BMI, body mass index; GA, gestational age; SGA, small for gestational age.

*** *p* < .01.

** *p* < .05.

* *p* < .1.

### 3.3 Subset and sensitivity analyses

Within the subsample of individuals with exposure levels under 5.0 pg/mg of albumin, we observed that a 1 unit increase in the natural log AFB1‐lysine is associated with a decrease in gestational age at delivery by 0.46 weeks (95% CI: −0.98, 0.067; *p* = .09), controlling for the same confounders as the previous analyses. Similar to the data shown in Table 3, we do not observe any statistically significant associations between our outcomes of interest and AFB1‐lysine levels in this subsample. We also did not find any effect modification between aflatoxin levels and wealth, age, education, height, and baseline BMI of the mother, sex of the child, and gestational age at enrollment, in the full sample or in the subsample (data not shown).

**Figure 2:**
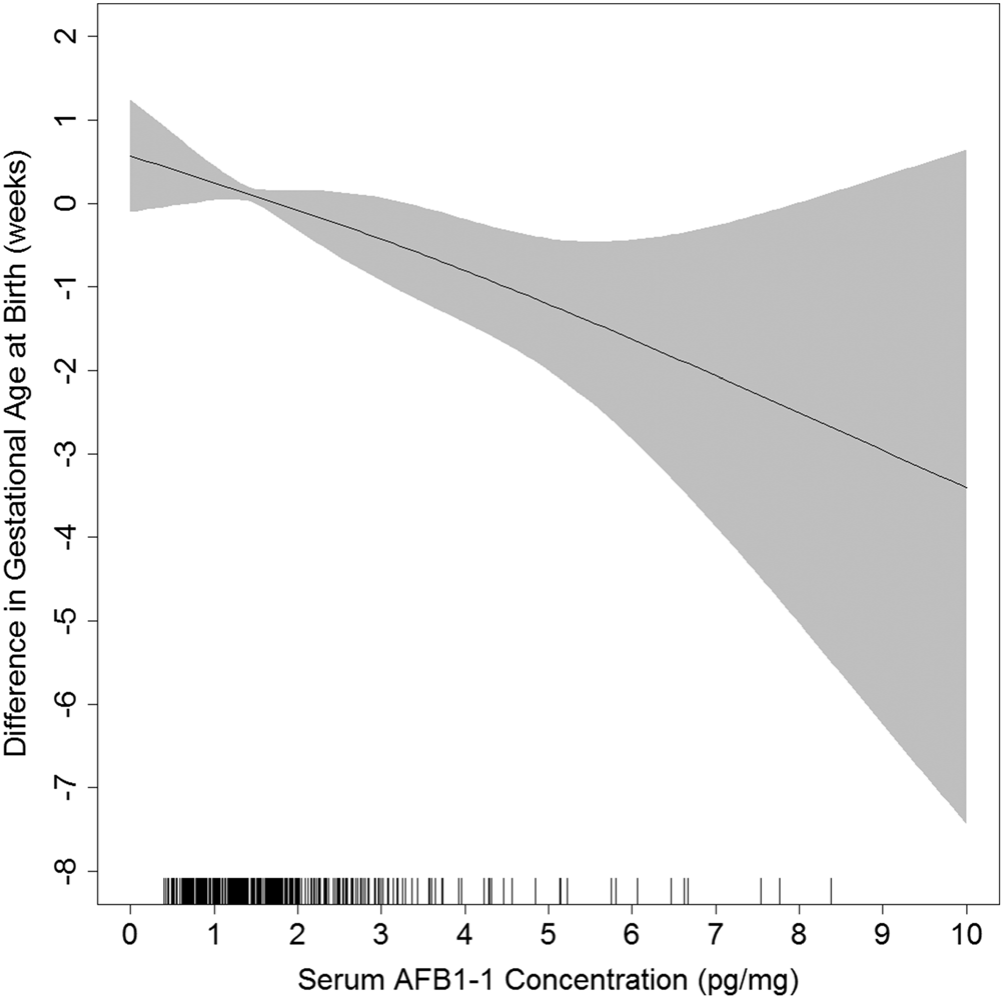
The same control variables were used as in the regression analyses reported in Table 3. The p‐value for the aflatoxin (AF) B1‐smooth term was 0.0066. The model only includes those with <10‐pg/mg AFB1‐lysine concentration

As a visual aid, the independent, potentially nonlinear association between serum AFB1‐lysine concentration and gestational age at birth within the low AFB1‐exposure subset is plotted in Figure 2. Based on this plot, the effects of aflatoxin plasma concentrations do appear to have a linear relationship with gestational age at birth.

When testing whether the effect of aflatoxin exposure on birth weight was mediated by gestational age at delivery, no statistically significant effects of the proportion mediated were observed. Additionally, we restricted our analysis to individuals in the upper and lower quartiles of AFB1‐lysine levels to observe whether there were differential relationships at the extremes; in this analysis, the coefficient for gestational age at delivery remains stable (−0.48; 95% CI: 0−.92, −0.03; *p* = .04). The *t*‐statistic for the effect of the natural log of AFB1 on birth weight decreases somewhat and trends negatively (−0.04; 95% CI: −0.16, 0.04; *p* = .31), and the effects on other outcomes remain stable.

## 4 DISCUSSION

Our findings from this urban cohort indicate lower levels of exposure than previous studies in East and West African rural populations. We find no associations between aflatoxin levels and infant growth or weight, and a small inverse relationship between aflatoxin plasma levels and gestational age at birth. Overall, the lack of association between in utero aflatoxin levels and birth weight is somewhat inconsistent with previous studies, showing growth inhibition in humans and animals from aflatoxin exposure, though results in the literature have been mixed.

There are several possible explanations for the absence of strong associations in our sample. One plausible explanation is that our study population was from an urban context with relatively low levels of exposure compared with previous studies, and a higher level of exposure may be required in order to observe adverse effects. The majority of research on this topic (with the exception of the Shuaib, Jolly, et al. study in Kumasi) has been conducted in rural agricultural populations, whereas our sample population lives in the capital city of Dar es Salaam and works in a nonagricultural setting. Our sample had a mean level of 2.4 pg/mg in women (range 0.0–84.8). These are substantially lower than AFB1‐lysine adducts observed in pregnant women in Kumasi, Ghana (mean 10.9 pg/mg; range 0.4–268.7; Shuaib, Jolly, et al., 2010) and in maternal blood (AF‐alb) during pregnancy in The Gambia (geometric mean: 40.4; IQR:4.8–260.8; Paul C. Turner et al., 2007) 1Note that the study in the Gambia was done using the ELISA method, which can report levels about 7–8 times higher than the method used in our study. These differences in the laboratory method used could have implications for the absence or presence of an effect of aflatoxin exposure on the outcomes being analyzed.. The levels found in our analysis are similar to a study of two cohorts in southwestern Uganda, using the HPLC‐fluorescence method; in one cohort of 713 individuals, 90% of samples were positive for AFB1‐lysine, with a median level of 1.58‐pg/mg albumin, whereas in the other, 92.5% of samples had detectable levels, with a median of 1.18‐pg/mg albumin (Kang et al., 2015). Their study also found that in one of the cohorts, agricultural and rural residents had higher levels of exposure compared with non‐rural, nonagricultural participants.

Urban populations tend to have a higher socioeconomic status, more diverse food environments, and markets with higher quality standards, all of which can support a more diversified diet that helps shield consumers from aflatoxin exposure (Kang et al., 2015; Liu & Wu, 2010). Dietary profiles are also markedly different in Dar es Salaam compared with other regions of Tanzania; in Dar es Salaam, rice and maize comprise about 20% of calories each. In other regions, maize accounts for approximately 40% of calories, rice an additional 10%, and the remainder of calories come from a smaller set of food categories as compared with Dar es Salaam (Cochrane & D'Souza, 2015). The lower contamination levels in rice relative to maize and the better dietary diversity likely contribute to the lower AFB1‐lysine levels observed in our study population and, consequently, fewer adverse health effects. Overall, dietary exposure to aflatoxins appears to be lower in Tanzania relative to other countries in sub‐Saharan Africa. For example, Liu and Wu (2010) report an aflatoxin exposure of 0.02–50 ng/kg of body weight/day in Tanzania, whereas neighboring Kenya has an exposure range from 3.5–133 ng/kg, the Gambia, 4–115 ng/kg, and Nigeria, 139–227 ng/kg (Liu & Wu, 2010). At these lower concentrations of exposure, the effects of AFB1‐lysine on pregnancy outcomes and growth may not be as severe.

Another possible explanation for the low levels of aflatoxin observed in this sample is due to the fact that this is a population of nonanemic, iron‐replete women based on the selection criteria for the original study (Etheredge et al., 2015). Smith et al. (2017) summarize the evidence showing the association between maternal aflatoxin concentrations and an increased risk of anaemia from one human study (from Shuaib, Jolly, et al., 2010); they describe several animal studies that show cytotoxicity and lysis of red blood cells at high levels of exposure, which may lead both anaemia and iron deficiency. In our sample, there could be selection bias wherein lower anaemia and iron deficiency may also be associated with lower aflatoxin exposure, leading to nongeneralizability of our results. Moreover, Smith et al. (2017) describe three pathways through which aflatoxin may influence birth outcomes: (a) through the mother, leading to maternal anaemia, and as a result, poor foetal growth and preterm birth; (b) through the placenta, leading to poor foetal growth; and (c) through the foetus, leading to miscarriage and stillbirth or preterm birth. By omitting the pathway of maternal anaemia caused by aflatoxin exposure, we may decrease the likelihood of its downstream consequences, which may explain the lack of association observed in many of our analyses. Our results could also suggest the importance of the maternal anaemia pathway, because there were fewer adverse effects observed in our sample when this pathway was omitted.

Lastly, another possibility is that aflatoxin exposure might not play as great of a role in child growth faltering as has been previously hypothesized. Findings from the first randomized trial designed to test the impact of a reduction in aflatoxin exposure on children's linear growth were recently published, also in an East African context (Hoffmann, Jones, & Leroy, 2018). This intervention improved access to aflatoxin‐free maize in Kenyan villages. Despite achieving reductions in serum AFB1‐lysine at end line, and some improvements in linear growth at midline, there were no differences in children's linear growth at end line. The results of a study by Chen et al. (2018) in Tanzania support our finding of the absence of an association between low‐level aflatoxin exposure and growth. In a cohort of 60 rural Tanzanian children ages 24–36 months in an area with a high prevalence of stunting (>75%), the authors found a mean AFB1‐lysine level of 5.1 pg/mg (Chen et al., 2018). They observed no association between weight‐for‐age and weight‐for‐height z‐scores and these relatively low plasma aflatoxin levels, although they did find a relationship between fumonisin exposure and underweight. These results suggest that low levels of aflatoxin may not contribute to growth impairment in this sample, though fumonisin may play a role (Chen et al., 2018). Leroy et al. found that low‐level aflatoxin exposure (median level = 0.82 pg/mg) was actually associated with greater linear growth in a longitudinal study of infants in southern Mexico (Leroy, Sununtnasuk, García‐Guerra, & Wang, 2018). Lastly, a recent study using a maternal and newborn cohort in Nepal found that relatively low maternal aflatoxin exposure was associated with a slight increased risk of being small for gestational age but not with birth weight, birth length, anthropometric z‐scores, low birthweight, stunting, or preterm birth (Andrews‐Trevino et al., 2019). Together, these findings suggest that the relationship between aflatoxin and child growth is more complex than previously understood. Aflatoxin exposure might not be a strong independent driver of child growth. Rather, it could be confounded by other factors like socioeconomic status (Leroy, Wang, & Jones, 2015), or operate through multiple pathways that are not well understood, like immunomodulation, inflammation, and environmental enteropathy (Smith, Stoltzfus, & Prendergast, 2012).

However, several other studies have found significant adverse effects of aflatoxin exposure in utero and birth outcomes and/or child growth. Four of the five studies reviewed by Shuaib, Ehiri, et al. (2010) found that aflatoxin exposure was inversely associated with birth weight, but for two of these studies, the association only held for female infants, and all of the study designs were cross‐sectional (Shuaib, Ehiri, et al., 2010). The study by Turner et al. of 107 infants in The Gambia linked aflatoxin exposure in utero, as measured through maternal, cord, and infant blood, to growth faltering in children based on anthropometry at 1 year of follow‐up (Paul C. Turner et al., 2007). The authors find that aflatoxin albumin levels in maternal blood were a strong predictor of both the infant's height and weight, estimating that a reduction in AF‐alb levels from 110 to 10 pg/mg would lead to an increase of 0.8 kg in weight and 2 cm in height within the first year of infancy. Placental weight and gestational duration were included among the confounders, and these could be important mediators of the effect of aflatoxin on growth. The study by Shuaib, Jolly, et al. found a statistically significant increase in the odds of low birth weight in the highest compared with the lowest quartiles of AFB1‐lysine levels (adjusted OR: 2.09; 95% CI: 1.19–3.68), but no significant association was observed for the outcomes SGA, preterm, or stillbirth. Although the sample size was higher than that of our study (785 pregnant women) and the higher exposure levels might have made effects more detectable, the design was cross‐sectional, such that blood samples were taken at delivery (Shuaib, Jolly, et al., 2010).

We observed a small inverse relationship between increases in AFB1‐lysine levels and gestational age at birth. To our knowledge, the only study examining the effect of aflatoxin exposure on gestational age measured AFM1 levels in breast milk and found no significant correlation between the presence of aflatoxin and postnatal age, gestational age, gender, and clinical condition of the infant (Abdulrazzaq, Osman, Yousif, & Al‐Falahi, 2003). It is always possible that this result is an artefact of multiple testing. The potential mechanism by which aflatoxin could lead to decreased gestational age has not been closely studied but could be a result of repeated insults to the foetus which may promote early labor through the release of inflammatory cytokines and waste products. In animals, aflatoxin has been associated with decreased live births, foetal abnormalities, and reduced litter size (Smith et al., 2017). The teratogenic effects in animals studies are thought to occur due to aflatoxin's ability to bind deoxyribonucleic acid and consequently inhibit or modulate protein synthesis (Smith et al., 2017). In a cohort of infants in The Gambia, Watson et al. investigated whether the inverse relationship they observed between lnAF‐alb and length‐for‐age, weight‐for‐age, and weight‐for‐length z‐scores could be explained by insulin‐like growth factor axis proteins, but found it could not (Watson et al., 2018). Though the effect size in our study was small, the potential effects of aflatoxin on gestational duration could have important public health implications for populations with higher levels of exposure, because early births can indicate the disruption of optimal prenatal health and initiate a cascade of suboptimal child growth and development.

Analyses of the relationship between exposure to aflatoxin and birth outcomes are complicated by three main methodological issues, making it difficult to compare across studies. First, the vast majority of studies have been cross‐sectional, making it impossible to determine a causal link and the time integration of the exposure in relation to the outcome. Second, these studies have used a number of different body fluids (including umbilical cord, maternal serum, and breast milk) and a number of different metabolites (e.g., B_1_ and M_1_), so results cannot always be compared (Shuaib, Ehiri, et al., 2010). Lastly, different researchers use different methods of sample analysis. Some have argued that the HPLC method for detecting aflatoxin levels is less sensitive than the ELISA and liquid chromatography with tandem mass spectrometry methods (McCoy et al., 2008), and Yard et al. (2013) found that the estimated AFB1‐lysine adduct values from the ELISA method are typically 4.6 times higher than those from the HPLC‐fluorescence measurement (Yard et al., 2013). As a result, aflatoxin levels differing across studies may also stem from differences in the laboratory method used, which should be noted when interpreting the results. Although there is still some debate about which method is best, the HPLC‐fluorescence method used in our study has been adopted by the Food and Drug Administration, United States Department of Agriculture, and Association of Official Agricultural Chemists for food and beverage analysis. Its recent wide use by researchers on this topic has allowed for an increased ease of comparisons across studies.

### 4.1 Strengths and limitations

Our analysis was subject to several limitations. First, blood samples and outcome measures were available for a nonrandom subset of participants, which may have introduced selection bias. We would expect these biases to be towards the null, because sample and outcome measure availability were likely unrelated to aflatoxin status. Our study design is observational, meaning that aflatoxin exposure might be associated with confounding factors, and is not randomly distributed in the population. To address this to the best of our ability, we conducted subset and sensitivity analyses and controlled for all known measured confounders. In addition, our study sample is not representative of the population of pregnant women in Dar es Salaam, because it selects for women who could access and give birth in a hospital and who were iron‐replete, human immunodeficiency virus‐negative, and nonanemic. Lastly, the effects of aflatoxin on intrauterine growth and birth outcomes are not well understood in relation to the time integration of exposure. It is possible that aflatoxin exposure has different effects on pregnancy outcomes depending on when the foetus is exposed. To try to address this, we controlled for gestational age at the time that the sample was taken.

This study also had several notable strengths. First, the laboratory team that analyzed the samples was blinded to the outcomes and data analysis process. Second, the longitudinal design allowed for a stronger causal interpretation of the effect of aflatoxin exposure on the outcomes. Lastly, data were collected at multiple time points in hospital facilities, which helped to ensure high quality and completeness of data through delivery.

## 5 CONCLUSION

Aflatoxin exposure has long been known to be a public health threat for carcinogenic reasons, and new evidence has emerged regarding its potential effects on growth faltering. This study contributes to a limited body of longitudinal evidence on the levels and effects of AFB1 exposure in East Africa, and specifically in an urban population in Dar es Salaam. More generally, our findings support similar recent research describing an unclear relationship between aflatoxin exposure and child birth and growth outcomes, especially at low levels of exposure.

Even if its effects on birth outcomes and child growth remain unclear, aflatoxin exposure is still a substantial public health threat due to its carcinogenic properties. In our study sample, the 99% prevalence of aflatoxin exposure at relatively low levels highlighted critical research gaps that need to be filled. Researchers should investigate potentially safe levels of exposure in utero, methods and timing of exposure measurement, the complex and interacting pathophysiology of multiple mycotoxins, and exposure management at the population and individual levels. In parts of Africa, South Asia, and Southeast Asia, chronic, low levels of aflatoxin exposure have been shown to be widespread. Although these health effects could be less obvious compared with higher exposure levels, the public health implications for such a large population could be substantial. The growing knowledge surrounding the extent of aflatoxin exposure and its health effects should be used to demonstrate the necessity of both improved food safety measures and expanded research into less understood areas.

## CONFLICTS OF INTEREST

The authors declare that they have no conflicts of interest.

## CONTRIBUTIONS

SB and WF prepared the initial proposal and designed the study. SA and FM facilitated the data collection. AMD managed and coordinated the data and assisted with the study and analysis design. JSW led the laboratory analysis. SP and SB analyzed the data. SP wrote the manuscript, with input from SB and JSW. SB, SA, AMD, FM, JKG, and WF edited the manuscript. All authors have read and reviewed the final manuscript.

## Supporting information

Data S1: Additional outcomes analyzed
