# Supplementary material for: Aflatoxin exposure in utero and birth and growth outcomes in Tanzania
Source: Matern Child Nutr. 2019 Dec 11;16(2):e12917. doi: 10.1111/mcn.12917 (PMC7083471; doi:10.1111/mcn.12917)
Supplement: Supplementary file 1 — Data S1: Additional outcomes analyzed [file MCN-16-e12917-s001.docx]

## Supplement

Appendix 1: Additional Outcomes Analyzed

|  | (1) | (2) | (3) | (4) | (5) |
| --- | --- | --- | --- | --- | --- |
| VARIABLES | Placental weight | Hb at delivery, g/dl | LAZ | WAZ | WLZ |
|  |  |  |  |  |  |
| ln of AFB1-lysine, pg/mg albumin | 5.97 | 1.08 | -0.0211 | -0.0486 | -0.0943 |
| 95% Confidence Interval | -14.50, 26.43 | -0.24, 0.38 | -0.34, 0.30 | -0.38, 0.28 | -0.46, 0.27 |
| p-value of coefficient | 0.57 | 0.65 | 0.90 | 0.77 | 0.61 |
| Constant | 479.4* | 7.050* | -9.810*** | -7.736** | 6.070 |
|  |  |  |  |  |  |
| Observations | 266 | 267 | 185 | 220 | 183 |
| R-squared | 0.079 | 0.083 | 0.095 | 0.111 | 0.064 |
| Multivariate regressions control for asset quintile, employment category, age of mother, sex of child, education category, treatment regimen, baseline body mass index of mother, average height of mother across all prenatal appointments, and gestational age at enrollment. *** p<0.01, ** p<0.05, * p<0.1. Coefficients and 95% confidence intervals are reported. Follow-up appointment data were available for 381 mothers, but not all follow-up appointments had associated anthropometry for the infant. Three, six, and four observations were excluded for LAZ, WAZ, and WLZ respectively, for being outside of the range of biologically plausible values (per WHO standard of a z-score >6 or <-6). The duration of follow-up ranged from 0.6 months to 25 months. Hb=hemoglobin; LAZ=length-for-age z-score; WAZ=weight-for-age z-score; WLZ=weight-for-length z-score. | | | | | |
